# Optimization of a Patient-Specific External Fixation Device for Lower Limb Injuries

**DOI:** 10.3390/polym13162661

**Published:** 2021-08-10

**Authors:** Mohammed S. Alqahtani, Abdulsalam Abdulaziz Al-Tamimi, Mohamed H. Hassan, Fengyuan Liu, Paulo Bartolo

**Affiliations:** 1 Mechanical Engineering Department, College of Engineering, King Saud University, Riyadh 11451, Saudi Arabia; 2 School of Mechanical, Aerospace and Civil Engineering, The University of Manchester, Manchester M13 9PL, UK; mohamed.hassan@manchester.ac.uk; 3 Industrial Engineering Department, College of Engineering, King Saud University, Riyadh 11451, Saudi Arabia; aaaltamimi@KSU.EDU.SA; 4 Department of Mechanical Engineering, School of Civil, Aerospace and Mechanical Engineering, Faculty of Engineering, University of Bristol, Bristol BS8 1TR, UK; fengyuan.liu@bristol.ac.uk

**Keywords:** additive manufacturing, design techniques, external fixation, topology optimization

## Abstract

The use of external fixation devices is considered a valuable approach for the treatment of bone fractures, providing proper alignment to fractured fragments and maintaining fracture stability during the healing process. The need for external fixation devices has increased due to an aging population and increased trauma incidents. The design and fabrication of external fixations are major challenges since the shape and size of the defect vary, as well as the geometry of the human limb. This requires fully personalized external fixators to improve its fit and functionality. This paper presents a methodology to design personalized lightweight external fixator devices for additive manufacturing. This methodology comprises data acquisition, Computer tomography (CT) imaging analysis and processing, Computer Aided Design (CAD) modelling and two methods (imposed predefined patterns and topology optimization) to reduce the weight of the device. Finite element analysis with full factorial design of experiments were used to determine the optimal combination of designs (topology optimization and predefined patterns), materials (polylactic acid, acrylonitrile butadiene styrene, and polyamide) and thickness (3, 4, 5 and 6 mm) to maximize the strength and stiffness of the fixator, while minimizing its weight. The optimal parameters were found to correspond to an external fixator device optimized by topology optimization, made in polylactic acid with 4 mm thickness.

## 1. Introduction

Musculoskeletal disorders and bone diseases resulting from aging or traumatic problems due to car accidents, wars and natural disasters represent one of the major health concerns in the world [1,2]. Bone fractures can be treated conservatively or using internal and/or external fixators depending on how extensive the damage is. Internal fixators such as fixation plates and intramedullary rods or nails are made in biocompatible metallic alloys and thus prone to some stress shielding problems [3]. Other clinical strategies include biological grafts (e.g., autografts and allografts), or synthetic bone graft substitutes produced using a wide range of biocompatible and usually biodegradable materials [4]. External fracture fixation is a common and well-established method used by orthopedic surgeons to treat musculoskeletal injuries [5,6,7]. This method is widely used for the treatment of different injuries or conditions such as compound fractures, acute fractures, soft tissue injuries, non-unions, delayed union, mal-union, and limb lengthening [8,9].

The concept of external fixation was proposed as an alternative immobilization method to plaster casts, internal fixation, and traction [10]. External fixation devices require the use of pins/wires that are needed to be inserted into the tissue and bone and then secured to the external part to provide support and to hold the broken bones in a proper position [11].

Various types of external fixators were developed for specific types of fractures, including planar, circular and hybrid external fixators [12]. These different external fixators, designed with different stiffness and stability characteristics, can be classified according to their frame configurations as unilateral, bilateral, quadrilateral, triangular, semi-circular, and circular [13,14,15]. The stiffness of the external fixators is critical to heal the fracture quickly and properly [9,16], but the optimal stiffness has not been identified yet. A more rigid frame can lead to a non-union or a delayed union, while a more flexible frame can lead to mal-union, non-union or pin–bone interface issues [17].

The mechanical performance of external fixation devices has been investigated by finite element analysis (FEA), considering either individual components of the frame or the entire assembled frame [16,18,19,20]. Sternick et al. [21] used FEA to study the relationship between the stiffness of the external fixator and the number of pins and concluded that the stiffness of the fixator with four pins is 19% and 42% higher than the stiffness of external fixators with three and two pins. Elmedin et al. [22] used FEA and experimental testing to analyze the stiffness of the Sarafix external fixator applied to a tibia with an open fracture under three loading conditions, axial compression, AP (anterior-posterior) four-point bending and torsion. The authors found that the Sarafix device showed remarkable results when compared with other fixation devices of the same type [22]. These research studies focused on the performance of different configurations of the external fixator but not on the optimization of the fixator in terms of geometry, weight and materials used. None of these studies, for example, applied design of experiments (DoE) or topology optimization to design and optimize external fixation devices according to a desired performance. In such cases, DoE is a relevant technique as the optimization process requires the use of multiple parameters and the understanding of their correlations. However, despite being used for a wide range of industrial applications [23,24,25], DoE has not been used for the design and optimization of bone fixation devices.

DoE is a statistical tool including a set of techniques such as factorial design, fractional factorial design and response surface method that is widely used to investigate the relationships between the factors affecting a process and their effects on one or multiple outputs [26]. It is considered an efficient and cost-effective tool to determine these relationships and to optimize process parameters [27]. The combination of finite element analysis and DoE has the potential to determine the optimal design parameters of an external fixator allowing to reduce unnecessary experiments.

Although external fixation devices present some benefits (e.g., standard devices for bone fractures treatment), they still display shortcomings (e.g., pain, long recovery time, infection, heavy frame) that need to be addressed [10,13,28]. Moreover, these devices are prefabricated in specific sizes, affecting the patient comfort and healing process [29]. Additive manufacturing is the ideal technology to manufacture personalized medical devices and implants [30,31]. Additive manufacturing is the process of creating a physical object from a digital 3D model by joining materials layer by layer [32]. This is also the ideal technology to produce personalized lightweight optimized external fixation devices. A wide range of additive manufacturing techniques and materials have been investigated as reported in [7]. This paper is the first study to address the need to redesign and develop a personalized (i.e., custom-fit) external fixator for lower limb injuries combining design optimization to be produced by additive manufacturing.

The aim of this study was to develop an integrated approach using a set of techniques including CT imaging, computer aided design (CAD), FEA and design methods to create a custom-made lightweight external fixator device aiming to be produced by additive manufacturing.

## 2. Modeling and Simulation

**Figure 1:**
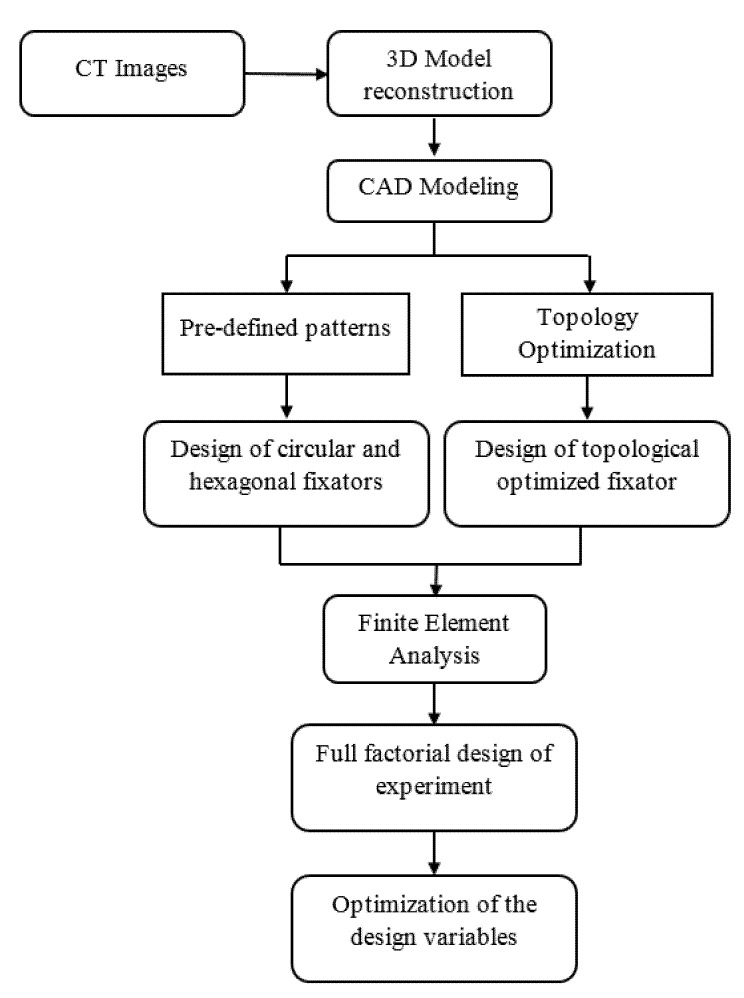
Methodology to design and optimization a personalized external fixation system.

The geometry and dimensions of the fixator were obtained from the anatomy of a 38-year-old female patient’s limb using computer tomography (CT) data obtained from Embodi3D (www.embodi3d.com, accessed on 1 August 2021). The CT-scanned data were exported as Digital Imaging and Communications in Medicine (DICOM) format into the 3D Slicer software version 4.10.1 (Harvard University, UK, www.slicer.org, accessed on 1 August 2021) to be converted to a Nearly Raw Raster Data (NRRD) format. This conversion was performed to visualize and transform the medical image data into a single file (single image) with a reduced size, guaranteeing the privacy of the patient’s data. The NRRD file was then uploaded into the democratiz3D software (Embodi3d platform, USA, www.embodi3d.com, accessed on 1 August 2021) to be automatically converted into a 3D solid STereoLithography (STL) model. Then, the STL model was exported to Solidworks 2018 (Dassault Systems, UK) for post-processing (e.g., checking errors, cleaning and removing unnecessary regions) and complete the design of the customized fixator, which was finally evaluated using finite element analysis (Ansys Workbench, Ansys, PA, USA). The main steps to design and evaluate the personalized external fixation device are presented in Figure 1.

**Figure 2:**
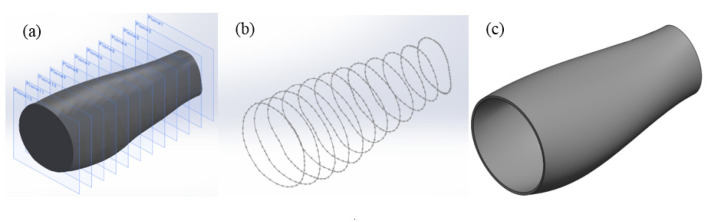
Key steps to obtain the final solid model. (**a**) Cutting planes; (**b**) splines; (**c**) loft, offset and thicken the surface.

In the Solidworks software, the area of interest was selected by creating two cutting planes at the boundaries of the imported model. Then, multiple cutting planes were created between the top and bottom planes due to the variation of the cross-section profile along the length of the leg (Figure 2a). Afterwards, multiple splines were created to represent the irregular shape of the leg at a specific position (Figure 2b). The number of considered planes has an impact on both design accuracy and processing time, which increase by increasing the number of planes. A loft operation was also performed to generate a smooth and continuous surface for modelling the external fixator. Considering the obtained surface as a reference, an offset surface operation was used to create a new surface allowing to specify the gap between the leg and the external fixator. This gap will be used to define a layer of a soft material aiming to reduce friction between the leg and the part, avoiding skin irritation and increasing patient comfort. This soft layer is not considered in this paper for simulation purposes. Afterwards, a thickness was specified to the offset surface in the outward direction, converting the surface into a solid model (Figure 2c).

The solid model was then split into three sections (top, middle and bottom). The optimization scheme will consider the middle section of the solid design, retaining the top and bottom sections to ensure the structural integrity of the fixator allowing also to integrate other elements such as wires and pins.

### 2.1. Design Techniques

Two different design techniques were used to create a lightweight external fixator: design with pre-defined patterns and topology optimization. The first method is based on the use of a pre-defined library of parametric shapes or patterns applied to the middle section of the model in a uniform distribution way. In this case, it is the responsibility of the designer to achieve a lattice structure with a proper performance enabling an adequate stress distribution. Contrarily, topology optimization corresponds to an automatic process to remove redundant material from the initial design without compromising its mechanical characteristics.

**Figure 3:**
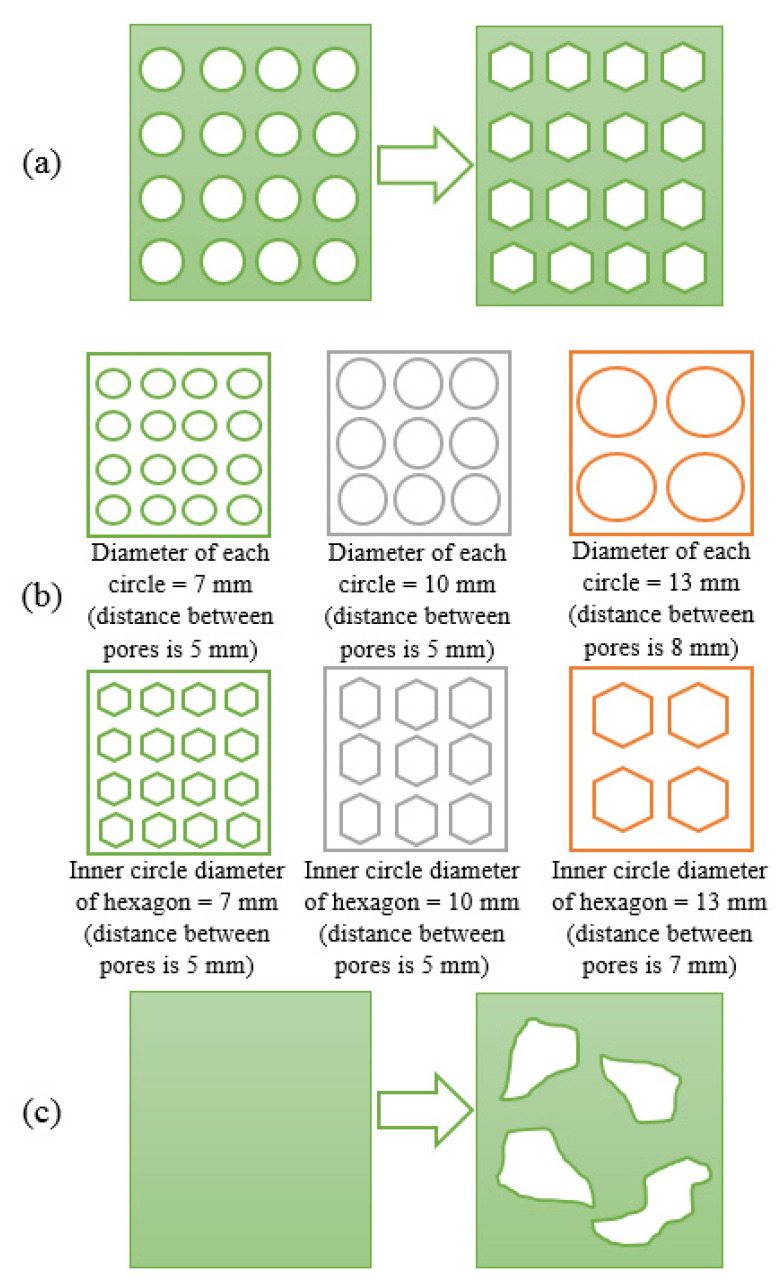
Considered case studies: (**a**) Case 1; (**b**) Case 2; (**c**) Case 3.

Three different case studies were considered:

- **Case 1:** the first method was used to investigate the geometric effect of two imposed patterns (circle and hexagon) on the performance of the fixator (Figure 3a);
- **Case 2:** investigates the effect of changing both the number and the geometric dimensions of the patterns considered in Case 1 for a fixed porosity value (Figure 3b). In this case, three different scenarios were also considered:
  ◦ **Case 2a—high number of patterned elements**: elements with 7 mm diameter.
  ◦ **Case 2b—middle number of patterned elements**: elements with 10 mm diameter.
  ◦ **Case 2c—low number of patterned elements**: elements with 13 mm diameter.
- **Case 3:** the middle section of the external fixator was modified using topology optimization (Figure 3c).

All the above cases were investigated considering the same mass reduction (25%) imposed to the middle zone of the fixator. The aim is not to investigate the effect of mass reduction but to investigate the effect of different design strategies on the mechanical properties of the fixation device.

#### 2.1.1. Design with Pre-Defined Patterns

**Figure 4:**
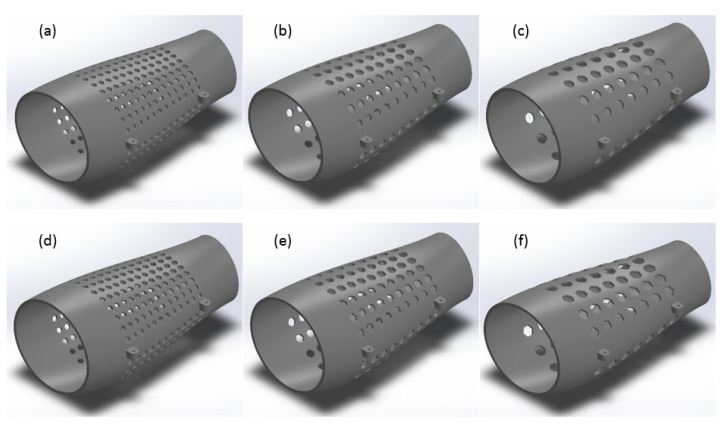
(**a**) Circular design with 7 mm diameter, (**b**) 10 mm diameter, and (**c**) 13 mm diameter; and (**d**) hexagonal design with 7 mm diameter, (**e**) 10 mm diameter, and (**f**) 13 mm diameter.

To create a porous structure with a predefined shape, construction sketches containing material removal parametric patterns were considered. The wrap command was used to create a porous external fixator design, and a fillet operation was then performed on all sharp corners (avoiding stress concentration areas). Obtained designs are presented in Figure 4. These different designs were then analyzed using the finite element analysis.

#### 2.1.2. Topology Optimization

Topology optimization is a mathematical approach that allows us to obtain the best material distribution within a given design domain according to a set of loading and boundary conditions [33,34]. Therefore, topology optimization allows us to design lightweight objects with an optimal mechanical performance. However, the optimized structure is often complex and requires refinement and simplifications using computer-aided techniques to allow the manufacturing of the part [35]. Topology optimization is widely used to solve a geometrical optimizing problem of minimizing the design compliance (i.e., strain energy), considering a specified volume/mass reduction value (design constraint), and can be mathematically formulated as follows [34]:

(1a) $$\min\;C:\;U^{T}F=\overset{N}{\underset{e=1}{\sum }}{\left[\rho_{e}\right]}^{p}{\left[u_{e}\right]}^{T}\left[k_{e}\right]\left[u_{e}\right]$$

$$\mathrm{subject}\;\mathrm{to}\;\{\begin{array}{cc} f=\frac{V}{V_{i}} & \;(1b)\\ F=\left[K\right]\times \left[U\right] & \;(1c)\\ 0<\rho_{min}\leq \rho_{e}\leq 1 & \;(1d) \end{array}$$

where C is the objective function, ρ(x,y,z) is the density of each element, *p* is the penalization factor, *f* is the volume fraction, *V* is the user-defined volume, *Vi* is the initial design volume and $k_{e}$ and $u_{e}$ are the element stiffness matrix and displacement vector, respectively. $\rho_{e}$ is the the relative density of the element e and $\rho_{min}$ is the minimum relative densities (non-zero for FEA stability).

The most common technique to solve the optimization problem is the Solid Isotropic Microstructure with Penalization (SIMP), which assumes a continuous function density as the design variable. This method has been employed for different applications such as structural [36], aerospace [37], architectural design [38] and medical applications [39,40,41]. The optimization process initiates by discretizing the design domain into a set of elements, assigning to each element a density (ρ) value of 0 or 1 (0 density means a void and 1 means a solid element) [40,41]. This method is controlled through the penalization factor. The governing equation that describes the SIMP process is given by the following equation [34]:

(2) $$E={\left(\rho_{e}\right)}^{p}E^{0}$$

where E is the Young’s modulus, and $E^{0}$ is the initial elastic modulus of the material (where ρ=1).

**Figure 5:**
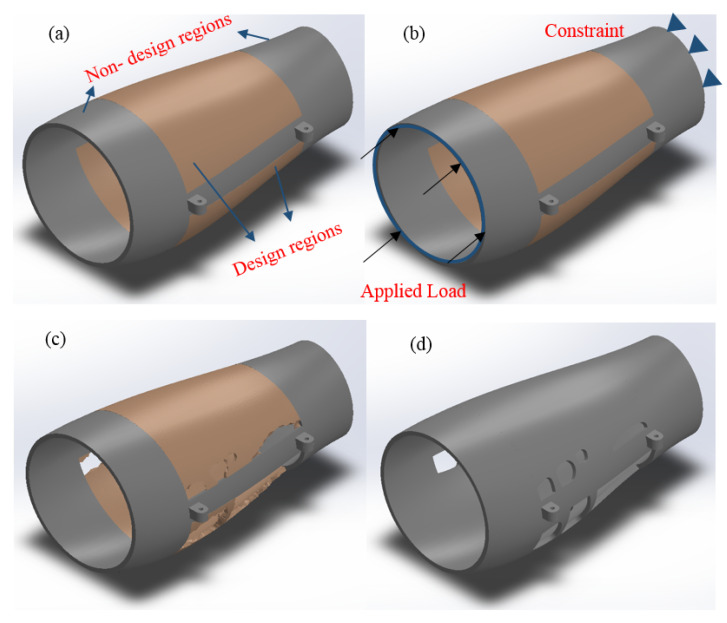
(**a**) Solid design, (**b**) loading and boundary conditions, (**c**) initial topology design and (**d**) final topology design.

In this work, the external fixator was topology optimized by imposing a mass reduction of 25% to the middle zone of the fixator (overall mass reduction of 13%) (Figure 5a). The optimization process was conducted assuming a uniaxial static compression load applied to one side of the fixator and fixing the opposite side (all displacements and rotations were assumed to be zero) (Figure 5b). The obtained topology-optimized model (Figure 5c) was refined, and the edges were smooth allowing us to obtain the final external fixator model (Figure 5d).

### 2.2. Structural Analysis of the External Fixator

All designs were numerically simulated (structural analysis) to evaluate their mechanical performance. Key structural parameters such as stresses, displacements, strength and stiffness of each design were evaluated. The strength and stiffness values were calculated as follows [42]:

(3) $$\mathrm{Fcritical}\;\left(\mathrm{strength}\right)=\frac{F\times Ys}{\sigma }$$

(4) $$K=\frac{F}{d}$$

where K is the compressive stiffness (N/mm), F is the applied force (N), and d is the corresponding displacement (mm). Fcritical (strength) is the load at the yield point (N), Ys is the yield strength of the material (MPa) and σ is the maximum Von Mises stress (MPa). All analyses were conducted following the standards for external skeletal fixation devices (ASTM F1541), which allows for part testing to be carried out without inclusion of the bone. Previous studies showed that the axial load is the most clinically significant load experienced by patients during the healing process once compared to the other loading conditions [43]. Therefore, all models were investigated under static uniaxial compression assuming a load magnitude of 700 N, which corresponds to the full weight of an average adult human (70 kg) [44]. This corresponds also to the worst-case scenario when the patient stands on only one leg. In all cases, the fixator was assumed to be fixed from the bottom (displacement and rotations were assumed to be zero) while the body weight was assumed to be applied from the top. The FEA software Ansys Workbench 19.2 (Ansys, Inc., Canonsburg, PA, USA) was used to conduct the structural analysis. Different parameters were investigated and optimized including frame thickness, materials to produce the device and the shape of holes. Three different polymeric materials commonly used in filament-based extrusion additive manufacturing and suitable for medical applications were selected: Polylactic acid (PLA), Acrylonitrile Butadiene Styrene (ABS), and Polyamide (PA) (Table 1).

Numerical simulations were performed considering a mesh of tetrahedral elements, and the different designs were assessed considering the maximum stress, displacement, and stiffness values. The same boundary conditions, load cases, mesh types and element sizes were applied to all models.

To reduce the number of design cases to be considered in the full factorial design study, a preliminary simulation was performed considering Case 2. Results, presented in Table 2, suggest that reducing the size of the elements and increasing the number of patterns yields a better performance and increases the strength and stiffness of the device. Therefore, only the designs corresponding to circle and hexagon patterns based on an inner circle diameter of 7mm were considered for further analysis.

### 2.3. Convergence Analysis

A convergence analysis was performed to determine the most suitable mesh size. Therefore, a series of simulations were performed considering different element sizes (5, 4, 3, 2, 1 mm). Based on the results, the meshes considered are the ones corresponding to an element size of 1 mm.

### 2.4. Full Factorial Design 

In this study, full factorial design was used to observe and identify any changes in the response due to the changes made to the variables [26]. This is a reliable and efficient approach due to its ability to discover the main effect of each factor as well as the interaction between all factors of the process, thereby achieving the optimal conditions [23,24,25].

Three design factors were considered: thickness, materials and fixator design. To ascertain the significance of each factor, several simulations were conducted at every combination of the design factor levels. The investigated factors and their corresponding level values are presented in Table 3. The working range for each design factor was identified as follows: four levels for thickness, three levels for the materials and three levels for the fixator design. The sample size required for this analysis is the product of the number of factor levels (4 × 3 × 3), which results in a total of 36 simulations. As previously reported [46,47,48], the thickness values of orthopedic devices fall within the range of 3–6 mm. The material factor was selected to be in three levels corresponding to ABS, PLA and PA. Finally, three levels were considered for the fixator design: circle, hexagon and topology optimized designs. The effects of two-level interactions (thickness*material, thickness*design and material*design) were also investigated. The three-factor interactions were eliminated as they are considered rare for high order interactions to be significant, and they are also difficult to interpret.

### 2.5. Statistical Analysis—Analysis of Variance (ANOVA)

ANOVA was used to test the following hypotheses:

**Hypothesis** **H01.** *Thickness, material and fixator’s design have no effect on the fixator’s measure*.

**Hypothesis** **H1.** *Thickness, material and fixator’s design influence the fixator’s measure*.

**Hypothesis** **H02.** *There is no interaction effect between thickness, material and fixator’s design on the fixator’s measure*.

**Hypothesis** **H2.** *There is an interaction effect between thickness, material and fixator’s design on the fixator’s measure*.

The Minitab 19 software (Minitab, USA) was used to obtain the ANOVA results. The analysis was carried out at the 5% significance level and 95% confidence level.

### 2.6. Performance Measures

Two different performance measures or responses (strength and stiffness) were selected to obtain a fixator with maximum strength and stiffness while reducing its weight. Numerical simulations were conducted considering 36 different settings, which represent all possible combinations of factors and levels to conduct the parametric analysis.

## 3. Results and Discussion

The design of an experiment matrix with the corresponding strength and stiffness are presented in Table 4. Analysis of variance (ANOVA) and the *p*-value were used to determine the significance of the design factors on the performance measures. Factors presenting a significant effect on the fixator’s strength and stiffness were examined through factorial plots: main effect plot and Pareto plot. The response optimizer tool was also used, aiming to obtain a strong, stiff and lightweight external fixator device.

### 3.1. ANOVA

ANOVA results are presented in Table 5 and Table 6. Results comprise the degree of freedom, sum of square, mean square, F-value and *p*-value. From Table 5, it is possible to observe that the *p*-value for all main factors and their two-level interaction are less than 0.05, which means that the main design factors and corresponding interactions are statistically significant. These results provided a strong evidence against the hypotheses **H01** and **H02**, which were consequently rejected. The ANOVA results for the stiffness performance measure are presented in Table 6. These results also show that the three main factors and their 2-way interactions are significant at the confidence level of 95%. Therefore, both **H01** and **H02** were rejected.

### 3.2. Main Effects

**Figure 6:**
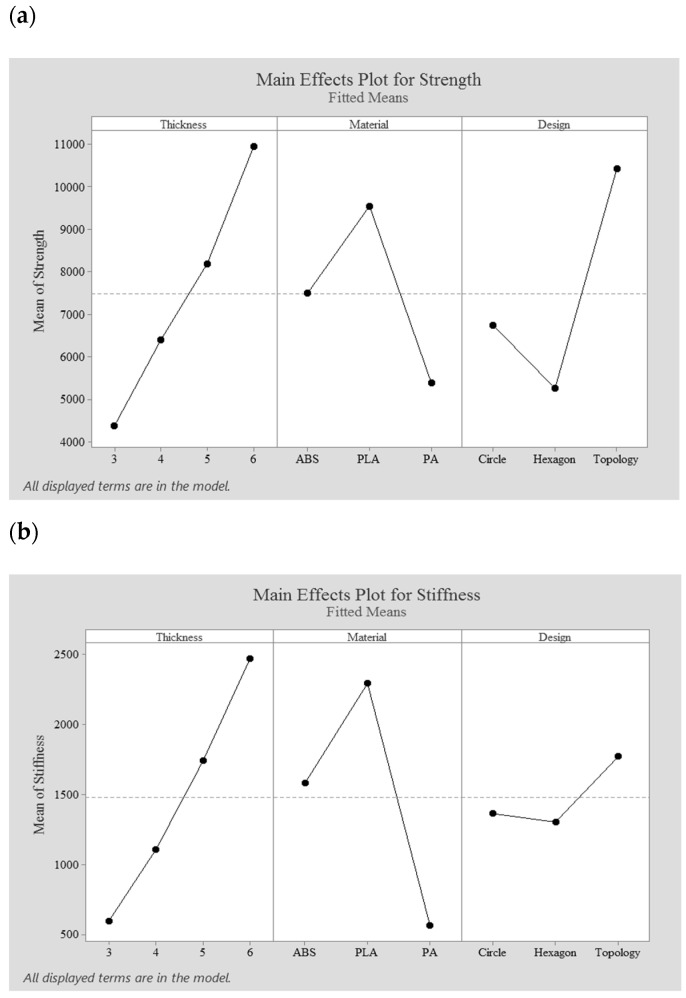
The main effect plot; (**a**) for the strength response and (**b**) for the stiffness response.

The main effects of each design factor on the strength and stiffness of the external fixator are shown in Figure 6. These plots represent the results of the regression analysis and only present the factors that are significant at the 95% confidence interval. Moreover, Figure 6 presents the main factors and shows how those factors increase or decrease the performance measure when the factor’s level changed. The mean of strength and stiffness factors at each level are plotted and connected by a line. The horizontal line indicates that the factor does not affect the performance measure, the presence of a slope indicates an effect, while a steeper slope means a greater magnitude of the effect. Therefore, from Figure 6, it was possible to determine which factor has the greatest effect on the performance measure.

From Figure 6, it can be observed that as the design factor changes from one level to another, the strength performance measure increases or decreases. The strength increases if the effect of the factor is positive when the factor’s level changes. In contrast, a reduction in the strength response occurs if the effect is negative. As observed, the effect of the thickness factor is positive, so an increase in strength is observed by increasing the thickness. The same trend is also observed for the stiffness performance measure. This trend was expected as the weight of the fixator increases by increasing the thickness of the fixator. For the material factor, the strength performance measure increases by changing the material level from ABS to PLA and decreases from PLA to PA, with a clear reduction in the fixator’s strength in the case of PA. The high strength of the PLA fixator is attributed to the high yield strength. Regarding the stiffness measure, it is also possible to observe that the ABS and PA fixators present lower stiffness values in comparison with the PLA fixators, due to the higher modulus of elasticity. Regarding the design factor and its effect on the strength and stiffness of the fixation device, results show that the effect is negative by moving from a circular to a hexagonal design. On the other hand, the strength and stiffness of the topology-optimized design increased by 54.6% and 29.8%, respectively, when compared to the circle configuration design. Results also show that topology optimized fixator exhibit an increase in strength (98.1%) and stiffness (35.8%) in comparison to the hexagonal design.

### 3.3. Pareto Chart

**Figure 7:**
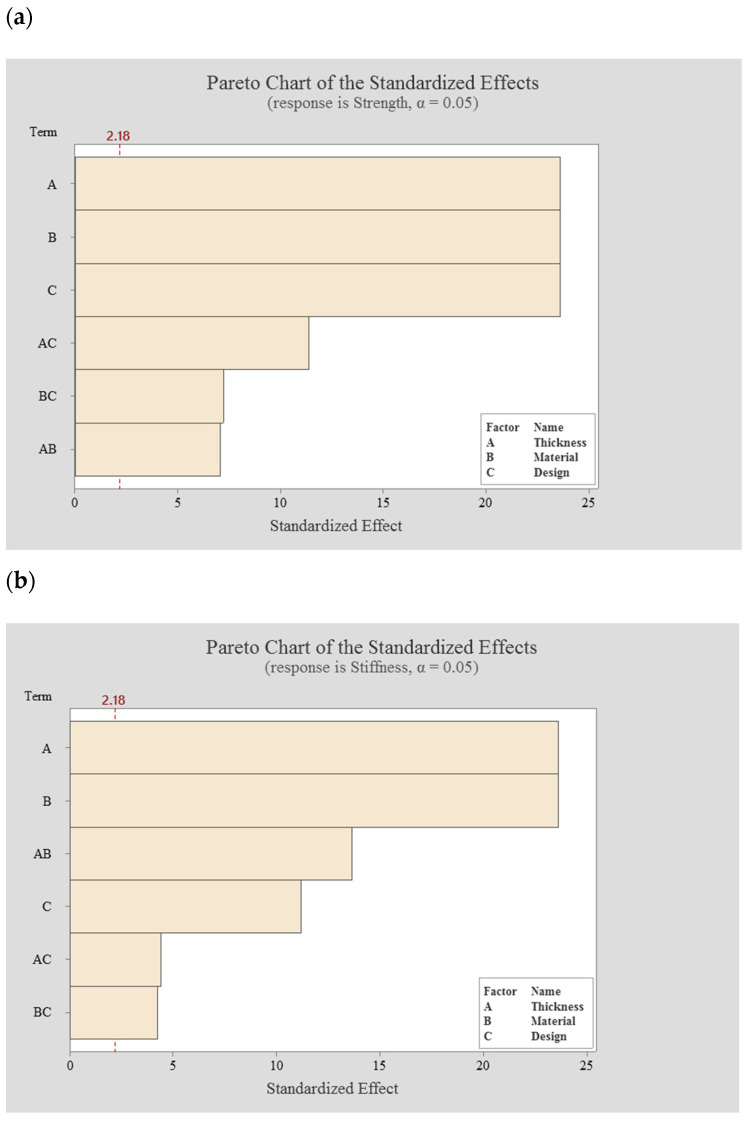
The Pareto chart; (**a**) for the strength response and (**b**) for the stiffness response.

Like the ANOVA, the Pareto chart is also a graphical tool that shows the significant factors and interactions that affect the performance measures. It gives the value of the effect and which effect is larger, showing the importance of each factor on its own and the two-level interactions of factors related to the strength and stiffness measures. Figure 7 shows the Pareto charts for both the strength and stiffness response. In this Figure, the horizontal column represents the values obtained from a Student’s *t*-test for each effect. The obtained t-value at the 95% confidence interval was 2.18. As shown in Figure 7, the magnitude values for the main factors and their interactions exceeded the reference value (2.18), which indicates the significance of these factors and their interactions on the strength at the level of 0.05. If the value is below the reference value, it can be considered not significant, and can be removed from the model for future predictions. From Figure 7a, it is possible to observe that the most significant factors on the strength are the thickness, material and design followed by the interaction between thickness and design. The importance of all main factors and interactions on the stiffness are also presented in Figure 7b. As observed, the thickness and material factors have a greater effect on the stiffness, while the design factor and all two-level interactions present smaller effects, being all significant at the 95% confidence interval. These results can be attributed to the effect of the materials’ Young’s modulus and the fixator’s thickness on the displacement, which affects the stiffness of the designed fixator.

### 3.4. Optimized Performance Measures 

Response optimizer in Minitab allows us to optimize the data, achieving a specific goal for multiple performance measures. In this study, the optimization was set to maximize strength and stiffness, and to minimize the weight. Based on the conducted simulations and specified objectives, the optimal design parameters, among all levels of factors, are:

- Fixator’s thickness: 4 mm
- Material: PLA
- Design: topology optimized fixator.

## 4. Conclusions

This paper presents a detailed procedure to design a novel type of external fixator. Different steps such as data acquisition (geometry of the human leg), data processing and modelling were described in detail. To reduce the weight of the fixator, different design strategies were considered (imposed patterns and topology optimization) and the mechanical performance was evaluated through FEA. Moreover, different parameters (thickness, material, geometry) were analyzed and optimized, and the influence of these parameters and their interactions on the strength and stiffness of the external fixator device was investigated using the ANOVA and the Pareto analysis. The optimal results were obtained through a response optimizer tool. Among the considered parameters, results showed that to obtain an external fixator that is strong, stiff and lightweight, the fixator should be made in PLA, with a thickness of 4 mm, and be topology optimized. Moreover, such a fixator is suitable to be produced using a filament-based extrusion additive manufacturing system.

Topology optimization results in a lightweight external fixation device with acceptable mechanical properties and better mechanical performance than the fixators designed by imposing pre-defined patterns. Moreover, it was found difficult to design the fixator considering pre-defined patterns due to the number of parameters that need to be investigated and optimized such as shape, size and number of holes. Additionally, results show that despite the simplicity of this method, topology optimization is the most effective method for mass reduction as it can remove the redundant material in an automatic way without compromising the mechanical performance. In the next phase of this research project, topology optimization will be considered to achieve high values of mass reduction without sacrificing the device’s functionality. Topology-optimized fixators will be additive manufactured and mechanically characterized for final validation.

## Figures and Tables

**Table 1:** Material properties [45].

	PLA	ABS	PA
Young’s Modulus (GPa)	2.35	1.62	0.58
Poisson Ratio	0.39	0.39	0.35
Yield Strength (MPa)	49.5	39	27.8
Density at 20 °C (gcm^−3^)	1.24	1.10	1.14

**Table 2:** Results of circular and hexagonal designs for different sizes. Results are based on external fixator devices made in PLA.

Design	Diameter (mm)	Von Mises Stresses (MPa)	Maximum Displacement (mm)	Strength (KN)	Stiffness (KN/mm)
Circle	7	4.75	0.438	7.28	1.59
	10	4.79	0.450	7.23	1.55
	13	4.84	0.452	7.15	1.54
Hexagon	7	6.34	0.458	5.46	1.52
	10	7.12	0.467	4.86	1.50
	13	7.79	0.474	4.44	1.47

**Table 3:** Main factors and their corresponding level values.

Factor	Number of Levels	Level Values			
		Level 1	Level 2	Level 3	Level 4
Thickness (mm)	4	3	4	5	6
Material	3	ABS	PLA	PA	-
Design	3	Circle	Hexagon	Topology	-

**Table 4:** Design of experiment matrix with the corresponding strength and stiffness.

Std Order	Thickness (mm)	Material	Design	Strength (KN)	Stiffness (KN/mm)
1	3	ABS	Circles	3.96	0.60
2	3	ABS	Hexagon	3.26	0.58
3	3	ABS	Topology	6.02	0.73
4	3	PLA	Circles	5.03	0.88
5	3	PLA	Hexagon	4.14	0.84
6	3	PLA	Topology	7.62	1.06
7	3	PA	Circles	2.84	0.22
8	3	PA	Hexagon	2.33	0.21
9	3	PA	Topology	4.25	0.26
10	4	ABS	Circles	5.73	1.10
11	4	ABS	Hexagon	4.31	1.05
12	4	ABS	Topology	9.36	1.40
13	4	PLA	Circles	7.29	1.60
14	4	PLA	Hexagon	5.46	1.53
15	4	PLA	Topology	11.81	2.02
16	4	PA	Circles	4.11	0.39
17	4	PA	Hexagon	3.06	0.38
18	4	PA	Topology	6.48	0.50
19	5	ABS	Circles	7.70	1.71
20	5	ABS	Hexagon	6.10	1.64
21	5	ABS	Topology	10.73	2.23
22	5	PLA	Circles	9.77	2.48
23	5	PLA	Hexagon	7.74	2.37
24	5	PLA	Topology	13.85	3.24
25	5	PA	Circles	5.49	0.61
26	5	PA	Hexagon	4.34	0.58
27	5	PA	Topology	7.98	0.80
28	6	ABS	Circles	9.73	2.41
29	6	ABS	Hexagon	7.54	2.30
30	6	ABS	Topology	15.56	3.21
31	6	PLA	Circles	12.35	3.50
32	6	PLA	Hexagon	9.55	3.34
33	6	PLA	Topology	19.93	4.65
34	6	PA	Circles	6.93	0.86
34	6	PA	Hexagon	5.31	0.82
36	6	PA	Topology	11.53	1.15

**Table 5:** ANOVA results for the strength performance measure.

Source	Degree of Freedom	Sum of Square	Mean Square	F-Value	*p*-Value
Model	23	525,641,896	22,853,995	273.54	0.000
Linear	7	482,345,330	68,906,476	824.75	0.000
Thickness	3	208,921,198	69,640,399	833.53	0.000
Material	2	103,717,907	51,858,954	620.71	0.000
Design	2	169,706,225	84,853,113	1015.62	0.000
2-Way Interactions	16	43,296,566	2,706,035	32.39	0.000
Thickness*Material	6	10,120,405	1,686,734	20.19	0.000
Thickness*Design	6	24,983,889	4,163,982	49.84	0.000
Material*Design	4	8,192,272	2,048,068	24.51	0.000
Error	12	1,002,579	83,548	-	-
Total	35	526,644,475	-	-	-

**Table 6:** ANOVA results for the stiffness performance measure.

Source	Degree of Freedom	Sum of Square	Mean Square	F-Value	*p*-Value
Model	23	42,226,405	1,835,931	191.79	0.000
Linear	7	37,313,541	5,330,506	556.86	0.000
Thickness	3	17,684,260	5,894,753	615.81	0.000
Material	2	18,079,921	9,039,961	944.38	0.000
Design	2	1,549,359	774,680	80.93	0.000
2-Way Interactions	16	4,912,865	307,054	32.08	0.000
Thickness*Material	6	4,055,959	675,993	70.62	0.000
Thickness*Design	6	5,00,306	83,384	8.71	0.001
Material*Design	4	356,600	89,150	9.31	0.001
Error	12	114,869	9572	-	-
Total	35	42,341,274	-	-	-

## Data Availability

The data presented in this study are available on request from the corresponding author.
